# Bone turnover and mineralisation kinetics control trabecular BMDD and apparent bone density: insights from a discrete statistical bone remodelling model

**DOI:** 10.1007/s10237-023-01812-4

**Published:** 2024-01-27

**Authors:** Natalia M. Castoldi, Edmund Pickering, Vittorio Sansalone, David Cooper, Peter Pivonka

**Affiliations:** 1 School of Mechanical, Medical and Process Engineering, Queensland University of Technology, Brisbane, Australia; 2 UMR 8208, MSME, Univ Paris Est Creteil, Univ Gustave Eiffel, CNRS, Créteil, France; 3 Department of Anatomy Physiology and Pharmacology, College of Medicine, University of Saskatchewan, Saskatoon, Canada; 4 Centre for Biomedical Technologies, Queensland University of Technology, Brisbane, Australia

**Keywords:** Bone turnover, Osteoporosis, Trabecular bone, Mineralisation kinetics, Bone mineral density distribution, Activation frequency

## Abstract

The mechanical quality of trabecular bone is influenced by its mineral content and spatial distribution, which is controlled by bone remodelling and mineralisation. Mineralisation kinetics occur in two phases: a fast primary mineralisation and a secondary mineralisation that can last from several months to years. Variations in bone turnover and mineralisation kinetics can be observed in the bone mineral density distribution (BMDD). Here, we propose a statistical spatio-temporal bone remodelling model to study the effects of bone turnover (associated with the activation frequency $\mathrm {Ac.f}$) and mineralisation kinetics (associated with secondary mineralisation $T_\textrm{sec}$) on BMDD. In this model, individual basic multicellular units (BMUs) are activated discretely on trabecular surfaces that undergo typical bone remodelling periods. Our results highlight that trabecular BMDD is strongly regulated by $\mathrm {Ac.f}$ and $T_\textrm{sec}$ in a coupled way. Ca wt% increases with lower $\mathrm {Ac.f}$ and short $T_\textrm{sec}$. For example, a $\mathrm {Ac.f}=$ 4 BMU/year/mm^3^ and $T_\textrm{sec}$ = 8 years result in a mean Ca wt% of 25, which is in accordance with Ca wt% values reported in quantitative backscattered electron imaging (qBEI) experiments. However, for lower $\mathrm {Ac.f}$ and shorter $T_\textrm{sec}$ (from 0.5 to 4 years) one obtains a high Ca wt% and a very narrow skew BMDD to the right. This close link between $\mathrm {Ac.f}$ and $T_\textrm{sec}$ highlights the importance of considering both characteristics to draw meaningful conclusion about bone quality. Overall, this model represents a new approach to modelling healthy and diseased bone and can aid in developing deeper insights into disease states like osteoporosis.

## Introduction

**Fig. 1:**
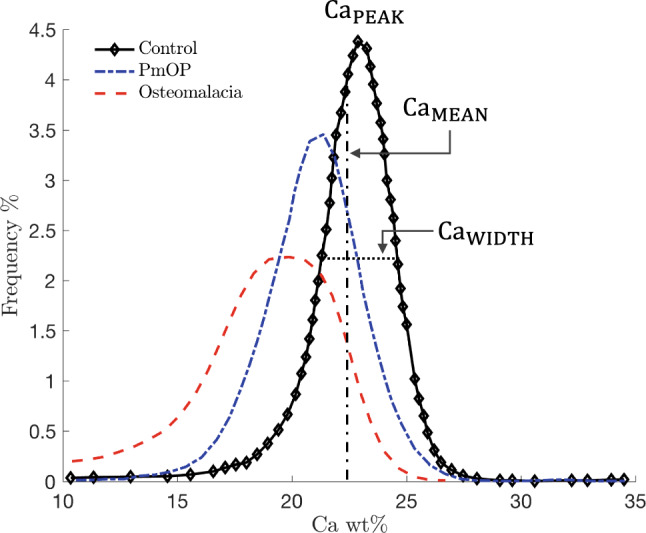
Comparison between BMDDs of trabecular bone from a healthy population (data from Roschger et al. (2008)), from a postmenopausal osteoporotic (PmOP) population, and from an osteomalacia population (data from Zoehrer et al. (2006)). $\mathrm {Ca_{PEAK}}$ indicates the peak in the distribution, $\mathrm {Ca_{MEAN}}$ the average degree of mineralisation, and $\mathrm {Ca_{WIDTH}}$ the width at half of the peak of the BMDD

Bone is a living tissue that undergo continuous repair, renewal, and adaptation to its biochemical and mechanical environment. The bone remodelling process is essential for removing microcracks accumulated in the bone matrix due to dynamic loading and to regulate mineral homeostasis and hematopoiesis (Parfitt 1994). This adaptive behaviour is achieved through the bone remodelling process, which is performed by the basic multicellular units (BMUs) as introduced by Frost (1969). The bone remodelling process on trabecular bone is activated at the bone surface, and concerted action of osteoclastic bone resorption followed by osteoblastic bone formation controls bone turnover (Parfitt 1984, 2002). During the bone mineralisation process the organic collagenous matrix deposited by osteoblasts becomes mineralised. The latter process is regulated by the mineralisation kinetics that exhibits two distinct phases: a fast primary mineralisation phase lasting for several days to a few weeks; and a secondary mineralisation phase that can last from several months to years (Bala et al. 2013). Given the nature of the mineralisation process, the mineralisation distribution depends strongly on the rate of bone turnover (Roschger et al. 2020), *i.e. *the number of BMUs being recruited, which can be described by the BMU activation frequency ($\mathrm {Ac.f}$). While bone diseases can affect different levels of bone structure, such as trabecular architecture in osteoporosis (Wehrli et al. 2001; Parfitt et al. 1983) and degree of mineralisation in osteomalacia (Roschger et al. 1998), the inhomogeneous mineral content and its spatial distribution on a microscopic scale are major determinants of the mechanical quality of trabecular bone (Roschger et al. 2008). The spatial heterogeneous distribution of mineral determines the bone mineral density distribution (BMDD) (Boivin and Meunier 2002, 2003). Roschger et al. (2020) defined the BMDD as the distribution of calcium content and described it as the “fingerprint” of bone, since it can distinguish healthy from pathological bone tissue at the scale of the bone matrix (Ruffoni et al. 2007) (see Fig. 1), regardless of skeletal site, sex or ethnicity (Roschger et al. 2003). Parameters that are typically extracted from the BMDD distribution curves are (i) calcium peak ($\mathrm {Ca_{PEAK}}$), *i.e. *the value of calcium weight percertage at the peak of the BMDD curve, (ii) calcium width ($\mathrm {Ca_{WIDTH}}$), *i.e. *width of the BMDD frequency curve at half of the peak of the BMDD, (iii) mean calcium weight ($\mathrm {Ca_{MEAN}}$).

Several mathematical approaches aim to include the effects of bone turnover and bone matrix mineralisation into models of bone remodelling. In the following, we review the three major types of models and outline their pros and cons.

1. Continuous spatial averaging approach: In these models the bone remodelling process is described for a representative volume element (RVE), *i.e. *a typical trabecular bone RVE $\approx$ 5 mm^3^ (Blöß and Welsch 2015). Usually, this approach formulates bone cell population models and the mineralisation process on this RVE while considering mechanobiological feedback (Martínez-Reina and Pivonka 2019; Martínez-Reina et al. 2021a). These types of models assume that several active BMUs in the RVE control bone turnover and density. However individual BMUs are not modelled explicitly, but only the accumulated action of osteoclasts and osteoblasts (of all active BMUs) control the changes in bone matrix volume fraction (or bone volume to total volume, known as BV/TV) and the average degree of mineralisation, *i.e. *ash fraction, in the RVE. These models have been typically applied in a pharmacokinetics-pharmacodynamics (PK-PD) modelling context to study the effects of different bone drug treatments on changes in BV/TV and mean mineralisation for patients with osteoporosis (Martínez-Reina et al. 2021a, b; Calvo-Gallego et al. 2022). We note that these models do not have the ability to recreate the BMDD and are not able to directly investigate the effects of changes in $\mathrm {Ac.f}$ and the spatial heterogeneity of the bone matrix.
2. Continuous BMDD balance approach: Ruffoni et al. (2007) described the changes in BMDD due to mineralisation as a “flow” from low to high values of the mineral content. They state that the area of the BMDD curve, *i.e. *calcium percentage by weight (Ca wt%) versus frequency in a constant bone volume, remains unchanged during the remodelling process. In fact, they conclude that the effect of remodelling on the BMDD is seen by a flow from lower Ca wt% values towards higher Ca wt% values (osteoblast action), while some bone volume is lost due to a “leakage” of the flow (osteoclast action). These effects were described using a BMDD balance equation in analogy with mass balance that can be mathematically expressed as a reaction-advection equation. Using this formulation, they investigated different mineralisation laws and their effects on BMDD in the RVE (Ruffoni et al. 2007). In subsequent work, Buenzli et al. (2018) extended this model to limit mineralisation to various maximum calcium capacities of bone. Results of this extended model show that an abrupt stopping of mineralisation near a maximum calcium capacity induces a pile-up of minerals in the BMDD statistics that is not observed experimentally. Using a smooth decrease in mineralisation rate, imposing low maximum calcium capacities helps to match peak location and width of simulated low-turnover BMDDs with experimental BMDDs. However, this study resulted in a distinctive asymmetric peak shape of the BMDDs (Buenzli et al. 2018). We note that even though these models are able to investigate the BMDD, no conclusions can be made regarding the bone microstructure.
3. Discrete statistical approaches: Martin (1984, 1991) showed that calculation of porosity and bone volume fraction are the same for two-dimensional (2D) and three-dimensional (3D) stereological approaches. In particular, discrete models allow us to calculate the porosity evolution, assuming perfect cylindrical osteonal remodelling perpendicular to the cortical cross section. Using discrete models of cortical bone remodelling Martin (1984, 1991) investigated various patterns of osteonal remodelling including overlap feedback, *i.e. *new secondary osteons have the tendency to remodel existing osteons, and random remodelling on the evolution of cortical porosity (Hazelwood et al. 2001; Nyman et al. 2004). Moreover, Heaney (1994); Heaney et al. (1997) used a discrete approach to model bone remodelling by assuming a certain number of BMUs in the RVE which could be integrated over a remodelling period to study the transient behaviour of bone remodelling. Later, Thomsen et al. (1994) developed a stochastic model of the remodelling process for human vertebral trabecular bone. Their simulations predicted the long-term effects of changes in the remodelling process on bone mass, trabecular thickness, and perforations of trabeculae. However, previous models do not include the mineralisation process and therefore cannot assess the calcium distribution.

Overall, even though the BMDD has great value to assess bone quality and identify bone diseases, the available bone remodelling models are not capable of recreating the spatial calcium heterogeneity and investigate the effects on BMDD and apparent density of factors driving bone remodelling. Therefore, we propose a discrete statistical two-dimensional computational model of the bone remodelling process. Based on the works of Martin (1984, 1991); Heaney (1994), this remodelling model of the trabecular bone is discrete both in space and time, but differently than previous discrete models, we include every phase of the remodelling process, from resorption to mineralisation. In this work, we focus on studying the effects of BMU $\mathrm {Ac.f}$ and mineralisation kinetics on the BMDD, as well as the bone microstructure. We apply this new approach to healthy and diseased bone, *i.e. *osteoporosis, and give insights on the contribution of bone remodelling factors on the overall bone quality.

## Discrete remodelling model for trabecular bone

All parameters used in the model can be found in Table 1, where symbols are based on the standardised nomenclature and symbols for bone histomorphometry (Dempster et al. 2013).

**Table 1:** Discrete bone remodelling model parameters

Symbol	Quantity
$\Omega _{RVE}$	Trabecular bone region
$\Omega _{\textrm{Bm}}$	Bone matrix region
$\Omega _{\textrm{Ma}}$	Marrow region
$\Omega _{\textrm{Rs}}$	Resorption region
$\Omega _{\textrm{F}}$	Formation region
$A_{RVE}$	Area of trabecular tissue
$A_{\textrm{Bm}}$	Area of bone matrix
$A_{\textrm{Ma}}$	Area of marrow
$f_{\textrm{Bm}}$	Area fraction of bone matrix
$f_{\textrm{Ma}}$	Area fraction of marrow
$\phi _{m}$	Area fraction of mineral
$\phi _{o}$	Area fraction of organic matter
$\phi _{w}$	Area fraction of water
$\rho$	Mean density of the bone matrix
$\rho _{m}$	Mineral mass density
$\rho _{o}$	Organic matter mass density
$\rho _{w}$	Water mass density
$\rho _{app}$	Apparent density
$\alpha$	Ash factor
${\bar{\alpha }}$	Mean ash value of the search region
$m_{\textrm{m}}$	Mineral mass
$m_{\textrm{dry}}$	Dry mass
$m_{\textrm{Ca}}$	Mass of calcium
$\mathrm {Ca\,wt\%}$	Calcium content
$\mathrm {Ca_{MEAN}}$	Mean value of the calcium content distribution
$\mathrm {Ca_{PEAK}}$	Peak value of the calcium content distribution
$\mathrm {B.Pm}$	Bone matrix perimeter
*p*	Position of a point $\in \Omega _{RVE}$
$\lambda$	Space resolution of *p*
$\textrm{Nb}_{RVE}$	Number of points $p\in \Omega _{RVE}$
*n*	Counter of remodelling events
$\mathrm {Ac.f}$	Activation frequency
$p_{\textrm{S}}$	Start BMU activation point
*t*	Time
*T*	Total simulation time
$\mathrm {Ac.P}$	BMU active time
$\mathrm {Rs.P}$	Resorption period
$\mathrm {Rv.P}$	Reversal period
$\textrm{FP}$	Formation period
$\textrm{Mlt}$	Mineralisation lag time
$t_{\textrm{F}}$	Time a new point is formed
$T_{\textrm{rep}}$	Periodicity of BMU activation events
$T_0$	Global time point: resorption starts
$T_1$	Global time point: resorption ends / reversal period starts
$T_2$	Global time point: reversal period ends / formation starts
$T_3$	Global time point: formation ends
$\tau _b$	Local time counter: follows evolution of active BMU
$\tau _m$	Local time counter: follows the mineralisation phase
$\tau _1$	Local time point: resorption ends / reversal period starts
$\tau _2$	Local time point: reversal period ends / formation starts
$\tau _3$	Local time point: formation ends
$R_{\textrm{On}}$	Hemiosteonal radium
${\bar{R}}_{\textrm{On}}$	Mean value of hemiosteonal radius
$\sigma _{\textrm{On}}$	Standard deviation of hemiosteonal radius
$r_{\textrm{Rs}}$	Resorption radius
$r_{\textrm{F}}$	Formation radius
$u_{\textrm{f}}$	underfilling coefficient
$\textrm{M}$	Mineralisation law
$c_1$	Mineralisation law parameter: calcium content
$c_{\textrm{max}}$	Mineralisation law parameter: calcium content
$t_1$	Mineralisation law parameter: characteristic time
$t_2$	Mineralisation law parameter: characteristic time
$T_\textrm{sec}$	Total time of secondary mineralisation

### Structure and composition of bone tissue

Let us consider a 2D region $\Omega _{RVE}$ of trabecular bone, small enough with respect to the whole trabecular compartment of the bone it belongs to. This region describes a slice of a Representative Volume Element (RVE) (around 5 mm^3^ in trabecular bone (Cowin 2001)). Hereinafter, following a stereological assumption, we will focus on a 2D description of bone and consider that our analysis is consistent with a 3D description. Therefore, bone composition will be described in terms of areas and area fractions of its constituents, that are assumed to be representative of their 3D counterparts–volumes and volume fractions, respectively.

At the tissue scale, trabecular tissue is made up of bone matrix and marrow, occupying the regions $\Omega _{\textrm{Bm}}$ and $\Omega _{\textrm{Ma}}$, respectively. It follows that:

1 $$\begin{aligned} A_{RVE}=A_{\textrm{Bm}}\left( t\right) +A_{\textrm{Ma}}\left( t\right) , \end{aligned}$$

where $A_{RVE}$, $A_{\textrm{Bm}}$, and $A_{\textrm{Ma}}$ are the areas of the whole trabecular tissue, and of the bone matrix and marrow regions, respectively; moreover, *t* denotes the time.

Equation (1) can be normalised with respect to $A_{RVE}$, and written in terms of area fractions:

2 $$\begin{aligned} 1=f_{\textrm{Bm}}\left( t\right) +f_{\textrm{Ma}}\left( t\right) \end{aligned}$$

where $f_{\textrm{Bm}}$ and $f_{\textrm{Ma}}$ are the area fractions of bone matrix and marrow space, respectively, at the tissue scale.

Let *p* be the 2D vector that denotes the position of a point in $\Omega _{RVE}$. Since our model is discrete in space and time, every point $p$ actually refers to a square pixel with side length equal to $\lambda$, here called the model resolution in space, and measured in $\mathrm {\mu }$m. All $p \in \Omega _{\textrm{Bm}}$ is considered to be part of the bone matrix, which basically consists of organic matter (mostly collagen), mineral, and water. Thus, neglecting the other minor constituents of bone matrix, the composition of each point of the bone matrix can be written as:

3 $$\begin{aligned} 1=\phi _{m}\left( p,t\right) +\phi _{o}\left( p,t\right) +\phi _{w}\left( p,t\right) , \end{aligned}$$

where $\phi _{o}$, $\phi _{m}$, and $\phi _{w}$ are the area fractions of organic matter, mineral, and water, respectively, at the pixel scale.

### Mass densities, ash factor, and calcium content

Let $\rho$ be the mass density of the bone matrix. It can be expressed as:

4 $$\begin{aligned} \rho \left( p,t\right) =\rho _{m}\phi _{m}\left( p,t\right) +\rho _{o}\phi _{o}\left( p,t\right) +\rho _{w}\phi _{w}\left( p,t\right) , \end{aligned}$$

where $\rho _{o}$, $\rho _{m}$, and $\rho _{w}$ stand for the mass densities of organic matter, mineral, and water, respectively. All these mass densities are assumed not to change over time.

For the sake of comparison with experimental observations, it is useful to compute the *apparent* mass density of bone matrix:

5 $$\begin{aligned} \rho _{app}\left( t\right) =\frac{1}{\textrm{Nb}_{RVE}}\,\sum _{p\in \Omega _{\textrm{Bm}}}\rho \left( p,t\right) , \end{aligned}$$

where $\textrm{Nb}_{RVE}$ is the number of points $p\in \Omega _{RVE}$.

The mineral content in the bone matrix is usually characterised by the ash fraction $\alpha$, which describes the ratio between the mineral mass $m_{\textrm{m}}$ and dry mass $m_{\textrm{dry}}$ (the sum of mineral and organic mass), reading:

6 $$\begin{aligned} \alpha \left( p,t\right) =\frac{m_{\textrm{m}}\left( p,t\right) }{m_{\textrm{dry}}\left( p,t\right) }=\frac{\rho _{m}\,\phi _{m}\left( p,t\right) }{\rho _{m}\,\phi _{m}\left( p,t\right) +\rho _{o}\,\phi _{o}\left( p,t\right) }. \end{aligned}$$

Provided the ash factor, computing the calcium content is straightforward. The calcium content is defined as the mass of calcium $m_{\textrm{Ca}}$ per dry mass, reading:

7 $$\begin{aligned} \mathrm {Ca\,wt\%}=\frac{m_{\textrm{Ca}}}{m_{\textrm{dry}}}=\frac{m_{\textrm{Ca}}}{m_{\textrm{m}}}\,\frac{m_{\textrm{m}}}{m_{\textrm{dry}}}=\frac{m_{\textrm{Ca}}}{m_{\textrm{m}}}\,\alpha =0.3989\,\alpha , \end{aligned}$$

where we dropped the dependency of the variables on space and time for the sake of clarity. The coefficient on the right-hand side of the above equation is obtained through the stoichiometric formula for hydroxyapatite. Hereinafter, we will denote $\mathrm {Ca_{MEAN}}$ and $\mathrm {Ca_{PEAK}}$ the mean and peak value of the calcium content distribution, respectively (Roschger et al. 2003) (see Fig. 1).

### Remodelling process

**Fig. 2:**
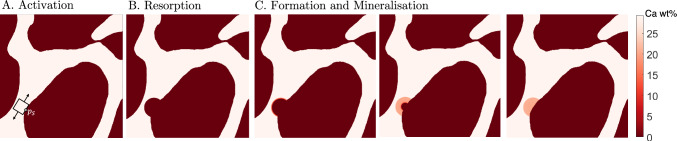
Diagram showing the development of a BMU on a cross section of trabecular bone $A_{RVE}$ (white and red represent bone and marrow, respectively): **A** Activation process giving position of a new BMU $\left( p_{\textrm{S}}\right)$ **B** resorption phase **C** three different stages of the formation and mineralisation stages which take place simultaneously

Let us assume that the 2D region $\Omega _{RVE}$ is experiencing remodelling and let $\mathrm {B.Pm}$ describe the bone matrix perimeter, *i.e. *the set of points belonging to the perimeter of $\Omega _{\textrm{Bm}}$, which is analogous to the trabecular surface in a 3D geometry. Remodelling is performed by basic multicellular units (BMUs), that are activated on $\mathrm {B.Pm}$ with a frequency $\mathrm {Ac.f}$. A remodelling event is depicted in Fig. 2. Remodelling starts with BMU activation at the point $p_{\textrm{S}}$ (Fig. 2A) and proceeds through the resorption (Fig. 2B), reversal (not shown), formation, and mineralisation (Fig. 2C) phases. First, we assume that the osteoclasts dig a semi-circular area of bone for a resorption period $\mathrm {Rs.P}$, as proposed by Parfitt (1994) in 1994. This is followed by a reversal period $\mathrm {Rv.P}$ in which the transition from resorption to formation takes place. Subsequently, we consider that osteoblasts lay down osteoid in semi-circular layers for a period $\textrm{FP}$. Finally, mineralisation takes place as new bone is laid down, after a mineralisation lag time $\textrm{Mlt}$. The mineral content increases during mineralisation, as mineral ions migrate from the interstitial fluid into the bone matrix to form hydroxyapatite ($\mathrm {Ca_{10}(PO_4)_6(OH)_2}$). These crystals nucleate within and outside the collagen fibrils, displacing the water present in the bone matrix (Bala et al. 2013).

**Fig. 3:**
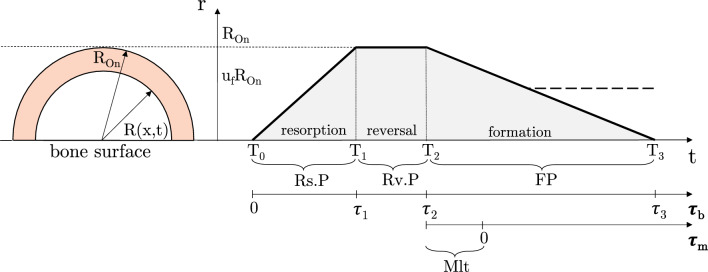
Hemiosteonal geometry and time course of the radius of the semi-circular region where remodelling takes place. Besides the global time axis *t*, local time axes $\tau _b$ and $\tau _m$ are introduced to follow the progression of the BMU and mineralisation, respectively. BMU is activated and resorption phase starts at local time $\tau _b=0$, corresponding to the global time $t=T_0$. Resorption phase ends and reversal phase starts at local time $\tau _1$, *i.e. *global time $T_1$. Reversal phase ends and formation phase starts at $\tau _2$, *i.e. *global time $T_2$. Formation phase ends at $\tau _3$, *i.e. *global time $T_3$–or earlier in case of unbalanced remodelling (dashed line)

The time course of the radius of the semi-circular region where remodelling takes place is depicted in Fig. 3. Each BMU is active during a time interval $\mathrm {Ac.P}=\mathrm {Rs.P}+\mathrm {Rv.P}+\textrm{FP}=T_3-To$. For each remodelling event, two *local* time counters are introduced: $\tau _b$ to follow the evolution of the active BMU across the activation, resorption, reversal, and formation phases; and $\tau _m$ to follow the mineralisation phase. We assume that osteoclastic bone resorption and osteoblastic bone formation are linear processes. The dashed line refers to unbalanced remodelling, namely when bone resorption prevails on bone formation–see Sec. 2.5.4 for more details.

It is important to note that we do not model the cellular dynamics related to bone remodelling. We assume that individual BMUs within the RVE have reached a steady-state configuration, *i.e. *all bone cell populations have reached their steady-state distributions (Buenzli et al. 2014).

### Remodelling algorithm

**Fig. 4:**
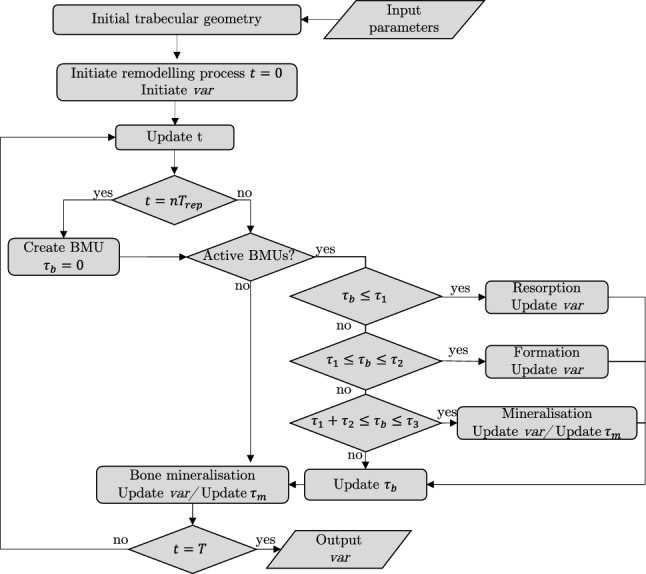
Discrete model iterative algorithm. After initiation of parameters and geometry, the simulation starts at time $t=0$. If *t* is equal to *n* times $T_{\textrm{rep}}$ (counter of remodelling events times the periodicity of BMU activation events) a new BMU is created and a local time counter $\tau _b$ starts to follow the evolution of the active BMU. Every BMU goes through every stage of the remodelling process. The duration of each event is given by the local time points ($\tau _1,\,\tau _2,\,\tau _2$), as explained in Fig. 3. Once a point *p* goes through formation it can be mineralised, and we initiate a mineralisation local time counter $\tau _m$ for every single formed point *p*. $p \in \Omega _{\textrm{Bm}}$ that do not belong to any active BMU and still have not attained the mineralisation threshold have their mineralisation level and mineralisation local time counter $\tau _m$ updated at the end of every iteration. Once all active BMUs and all bone have gone through their remodelling phase the time *t* is updated, and the process is repeated until *t* reaches the total simulation time *T*. The model output $\textrm{var}$ includes $f_{\textrm{Bm}},\,f_{\textrm{Ma}},\,\phi _{o},\,\phi _{w},\,\phi _{m},$ and $\mathrm {Ca\,wt\%}$

The iterative algorithm of our discrete model is shown in Fig. 4. For every simulation, we input the trabecular bone geometry from micro-CT images; the initial Ca wt% in the $\Omega _{RVE}$; the hemiosteonal geometry as described in Fig. 3; the mineralisation law; and the activation frequency $\mathrm {Ac.f}$ (see Table 2 for more details). From the $\mathrm {Ac.f}$ and total simulation time *T*, we identify the total amount of BMUs to be created. We initiate the simulation at time $t=0$. When *t* is equal to *n* times $T_{\textrm{rep}}$ (counter of remodelling events times the periodicity of BMU activation events) a new BMU is created and we initiate a local time counter $\tau _b$ that follows the evolution of the active BMU. While the BMU is active, it goes though every stage of the bone remodelling process: resorption, reversal, formation and mineralisation. At every time $\tau _b$ the output variables $\textrm{var}$ are updated, where $\textrm{var}$ includes area fractions and the calcium concentration ($\textrm{var}=\left[ f_{\textrm{Bm}},f_{\textrm{Ma}},\phi _{o},\phi _{w},\phi _{m}, \mathrm {Ca\,wt\%}\right]$). It is important to note that multiple BMUs can be active simultaneously, since each BMU holds their own local time counter $\tau _b$. Once a point *p* goes through formation it can be mineralised, and a mineralisation local time counter $\tau _m$ is initiated for every single formed point *p*. The mineralisation level evolves through a mineralisation law, described in Sec. 2.5.5. Points in $\Omega _{\textrm{Bm}}$ that do not belong to any active BMU and still have not attained the mineralisation threshold have their mineralisation level and mineralisation local time counter $\tau _m$ updated at the end of every iteration. Once all active BMUs and all bone have gone through their remodelling phase the time *t* is updated, and the process is repeated until *t* reaches the total simulation time *T*.

### Remodelling phases

The different phases of the remodelling process and their implementation in our model are described in this section.

#### Activation phase

The factors that initiate bone remodelling process are not completely understood, but BMU activation is believed to occur partly due to biomechanical demands, *e.g. *removal of microcracks from the bone matrix–hereinafter referred to as *targeted remodelling*–and due to biological demands, *e.g. *calcium and phosphate homeostasis–referred to as *random remodelling* (Wss 2001). In our model, the activation phase is governed by the *activation frequency* $\mathrm {Ac.f}$, *i.e. *the number of BMUs activated per unit time and unit bone area, that describes the intensity of remodelling. $\mathrm {Ac.f}$ is meant to be an input parameter of our model and is measured in BMU/mm^2^/year. The total number of BMUs activated during a time interval $\Delta t$ in the bone region $\Omega _{RVE}$ is given by:

8 $$\begin{aligned} N=\mathrm {Ac.f}\,\Delta t\,A_{RVE}. \end{aligned}$$

Our model can describe both random and targeted remodelling processes. In random remodelling, BMUs are randomly activated at any point of the boundary $\mathrm {B.Pm}$. In targeted remodelling, BMU activation is related to the mineralisation of the bone matrix, as microcracks are more likely to appear at bone sites which are highly mineralised. Hereinafter, we will focus on targeted remodelling.

Let *n* be a counter of remodelling events. We assume that remodelling events are evenly distributed in time, the periodicity being $T_{\textrm{rep}}=1/(\mathrm {Ac.f}\,A_{RVE})$. Thus, the *n*-th remodelling event starts at $t^n=n\,T_{\textrm{rep}}$. In order to activate the BMU, we search the $\Omega _{RVE}$ for the regions on the boundary of the bone matrix featuring the highest average degree of mineralisation. More precisely, for each $p\in \mathrm {B.Pm}$ we define a search region around $p$ as shown in Fig. 2A (the size of the search region is a model parameter) and compute the average value of $\alpha$ within it, to be called ${\bar{\alpha }}$. The activation point of the *n*-th BMU, referred to as $p_{\textrm{S}}^n$, corresponds to the region featuring the highest value of ${\bar{\alpha }}$. If several regions present the same value of ${\bar{\alpha }}$, $p_{\textrm{S}}^n$ is chosen randomly among those regions.

Eventually, the time counter $\tau _b^n$ is started and remodelling moves to next phase.

#### Resorption phase

Trabecular resorption may produce different patterns, *e.g. *trenches and pits. For the sake of simplicity, we assume resorption to produce hemiosteonal patterns (Parfitt 1994), *i.e. *semi-circular cavities with (hemiosteonal) radius $R_{\textrm{On}}$– see Fig. 2B.

The resorption phase of the *n*-th BMU takes place over the *local* time interval $[0..\tau _1]$, corresponding to the *global* time interval $[T_0^n..T_1^n]$. Bone resorption starts at $p_{\textrm{S}}^n$ (for $\tau _b^n=0$) and proceeds radially up to a distance $R_{\textrm{On}}$ from $p_{\textrm{S}}^n$ (for $\tau _b^n=\mathrm {Rs.P}$). Let $r_{\textrm{Rs}}$ be the current *resorption radius*, defining the limits of the current *resorption region*. We assume $r_{\textrm{Rs}}$ to evolve linearly in time, namely:

9 $$\begin{aligned} r_{\textrm{Rs}}\left( \tau _b^n\right) =\frac{R_{\textrm{On}}\,\tau _b^n}{\mathrm {Rs.P}}. \end{aligned}$$

The current resorption region is:

10 $$\begin{aligned} \Omega _{\textrm{Rs}}^n\left( \tau _b^n\right) = \left\{ \,p\,:\left\| \, p-p_{\textrm{S}}^n \right\| \le r_{\textrm{Rs}}\left( \tau _b^n\right) \,\right\} . \end{aligned}$$

#### Reversal phase

The reversal phase links bone resorption and bone formation. It sets the stage for the subsequent phases of bone remodelling, allowing for the deposition of new bone tissue. The coupling mechanisms between osteoclasts and osteoblasts responsible for generating an osteogenic environment remain poorly understood (Delaisse 2014). In our model, the reversal phase is a quiescent phase.

The reversal phase of the *n*-th BMU takes place over the *local* time interval $[\tau _1..\tau _2]$, corresponding to the *global* time interval $[T_1^n..T_2^n]$. Throughout this phase, the radius of the remodelling region is equal to $R_{\textrm{On}}$.

#### Formation phase

In the formation phase, osteoblasts lay down layers of unmineralised bone matrix called osteoid. Osteoid is composed of collagen and other proteins that provide the framework for mineralisation. Similarly to the resorption, in our model bone formation is assumed to proceeds radially from the hemiosteonal wall (*i.e. *the cement line) towards $p_{\textrm{S}}^n$–see Fig. 2C.

The formation phase of the *n*-th BMU takes place over the *local* time interval $[\tau _2..\tau _3]$, corresponding to the *global* time interval $[T_2^n..T_3^n]$. Bone formation starts at the cement line ($\tau _b^n=\tau _2$) and proceeds radially towards $p_{\textrm{S}}^n$ leading to complete ($\tau _b^n=\tau _3$) or incomplete ($\tau _b^n<\tau _3$) refilling of the resorption cavity. Let $r_{\textrm{F}}$ be the current *formation radius*, defining the limits of the current *formation region*. We assume $r_{\textrm{F}}$ to evolve linearly in time, namely:

11 $$\begin{aligned} r_{\textrm{F}}\left( \tau _b^n\right) =R_{\textrm{On}}\left( 1-\frac{\left( \tau _b^n-\tau _2\right) }{\textrm{FP}}\right) . \end{aligned}$$

The current formation region is:

12 $$\begin{aligned} \Omega _{\textrm{F}}^n\left( \tau _b^n\right) = \left\{ \,p\,:r_{\textrm{F}}\le \left\| \, p-p_{\textrm{S}}^n \right\| \le R_{\textrm{On}}\,\right\} . \end{aligned}$$

Just after being laid down, new bone is completely unmineralised ($\phi _{m}=0$). This new bone is basically a hydrogel made of collagen and water. The collagen content of bone can vary depending on factors such as species, age, diseases, anatomical location, and bone type (cortical or trabecular). However, variations are generally limited. The volume fraction of the organic matter is assumed to be the same for all the bone matrix points and not to change over time. The value $\phi _{o}=\frac{1}{3}$ has been used hereinafter (Vuong and Hellmich 2011; Martin 1984). Thus, in view of Eq. (3), the *initial* composition of each new point $p\in \Omega _{\textrm{F}}^n$ is set as:

13 $$\begin{aligned} \phi _{o}=\frac{1}{3}, \qquad \phi _{m}=0, \qquad \phi _{w}=\frac{2}{3}. \end{aligned}$$

Bone resorption and formation are balanced in a healthy remodelling process. By contrast, reduced bone formation can be associated with pathological bone remodelling. In our model, healthy remodelling corresponds to the case where the hemiosteonal cavity–dug during the resorption phase–is entirely filled during the formation phase. Accordingly, unbalanced remodelling can be simulated by considering that the hemiosteonal cavity is only partially filled during the formation phase. The formation radius at the end of the formation phase can be expressed as $u_{\textrm{f}}R_{\textrm{On}}$, with $u_{\textrm{f}}$ being the *underfilling coefficient*. The latter is a model parameter taking values between 0 (complete refilling of the hemiosteonal cavity) and 1 (no filling). We implemented partial refilling in our model by stopping the formation phase as soon as $r_{\textrm{F}}$ (as per Eq. (11)) drops below $u_{\textrm{f}}R_{\textrm{On}}$.

#### Mineralisation phase

Every point of bone matrix goes through the mineralisation process. Mineralisation of osteoid (laid down in the formation phase) starts after a *mineralisation lag time* $\textrm{Mlt}$, during which collagen fibres within the osteoid organise so as bone can form properly. In our model, after a new point $p\in \Omega _{\textrm{F}}^n$ is laid down at (global) time $t_{\textrm{F}}(p)$, an associated time counter $\tau _m$ is introduced to follow its mineralisation, and it is started after $\textrm{Mlt}$ from $t_{\textrm{F}}(p)$, *i.e.* $\tau _m(p,t)=t-\left( t_{\textrm{F}}(p)+\textrm{Mlt}\right)$.

Once mineralisation has started in the osteoid, a continuous increase in its mineral content occurs. Mineralisation is described by the evolution of the ash factor according to a mineralisation law $\textrm{M}(\tau _m)$, namely: $\alpha \left( p,t\right) =\textrm{M}\left( \tau _m\left( p,t\right) \right)$. As discussed in the introduction, primary mineralisation constitutes the first rapid phase of the mineralisation process. In this period, the ash factor is reported to reach the value $\alpha =0.45$ (Hernandez et al. 2000). The mineralisation process is then followed by a slow and gradual increase in the mineral content over a longer period of time, *i.e. *the secondary mineralisation, which evolves to a maximum Ca concentration of 30 wt% (Currey 2004; Ruffoni et al. 2007). We tested all the mineralisation laws proposed by Ruffoni et al. (2007) to represent both stages of mineralisation. The most promising BMDDs were obtained through the double exponential and hyperbolic mineralisation laws. For the sake of simplicity, we describe the mineralisation law through a double exponential curve:

14 $$\begin{aligned} \textrm{M}\left( \tau _m\right) =c_1\,\left( 1-e^{-\frac{\tau _m}{t_1}}\right) +\left( c_{\text {max}}-c_1\right) \,\left( 1-e^{-\frac{\tau _m}{t_2}}\right) , \end{aligned}$$

where $c_1$ and $c_{\text {max}}$ are calcium contents, and $t_1$ and $t_2$ are characteristic times–see App. 1 for more details. It is important to note that the total time of the secondary mineralisation is defined by $T_\textrm{sec}$, and the slope of the secondary mineralisation curve, *i.e. *second exponential, is given by $t_1$.

As mentioned in Sec. 2.5.4, the area fraction of the organic matter is assumed to be the same for all points and not to change over time. Thus, mineralisation of a bone matrix point emerges as a perfectly balanced increase of its mineral area fraction $\phi _{m}$ and decrease in water area fraction $\phi _{w}$. Using Eq. (6), the mineral area fraction can be written for each point $p\in \Omega _{\textrm{Bm}}$ as:

15 $$\begin{aligned} \phi _{m}\left( p,t\right) =\frac{\alpha \left( p,t\right) \rho _{o}\,\phi _{o}}{\rho _{m}-\alpha \left( p,t\right) \,\rho _{m}} . \end{aligned}$$

Eventually, since $\phi _{o}$ is known and $\phi _{m}(p,t)$ is provided by Eq. (15), Eq. (3) allows computing $\phi _{w}(p,t)$.

### Model implementation

The presented model was implemented and evaluated using MATLAB R2022a with suitable initial conditions to investigate changes in BMDD distribution due to variation in activation frequency and mineralisation kinetics.

#### Geometry

We define the $\Omega _{RVE}$ geometry from and in-silico generated trabecular bone as typically observed by micro-CT imaging. The trabecular bone generated image has a resolution of $1\mathrm {\mu }\textrm{m}\times 1\mathrm {\mu }\textrm{m}$ and trabecular thickness ranging from 200$\mathrm {\mu }$m to 400$\mathrm {\mu }$m (Liu et al. 2010). We use an edge detection tool implemented in MATLAB R2022a to identify the bone perimeter $\mathrm {B.Pm}$. The $\Omega _{RVE}$ is binarised into bone and marrow areas, and lacuno-canalicular porosity in bone matrix is neglected, which contributes about 3–5 % porosity (Cowin 1999; Ashique et al. 2017)

#### Initial state and model parameters

At the initial state, we assumed the ash factor uniform throughout $\Omega _{\textrm{Bm}}$ and equal to 70%. Model parameters related to the remodelling process are adapted from the literature and reported in Table 2. Note that the resorption radius $R_{\textrm{On}}$ is defined randomly at each new BMU initiation according to a normal distribution with mean value ${\bar{R}}_{\textrm{On}}$ and standard deviation $\sigma _{\textrm{On}}$. The total time of secondary mineralisation ($T_\textrm{sec}$) and the activation frequency ($\mathrm {Ac.f}$) are free parameters (*f.p.*) of our model and will be discussed afterwards. Eventually, the time step in all simulations is equal to 1 day.

**Table 2:** Input parameters for the discrete bone remodelling model. *f.p.*: free parameters

Input Parameters	Symbol	Values	Unit	Source
Resorption period	$\mathrm {Rs.P}$	21	days	Martin et al. (1998)
Reversal period	$\mathrm {Rv.P}$	10	days	Martin et al. (1998)
Formation period	$\textrm{FP}$	91	days	Martin et al. (1998)
Mineralisation lag time	$\textrm{Mlt}$	10	days	Martin et al. (1998)
Hemiosteon radius distribution: mean value	${\bar{R}}_{\textrm{On}}$	100	$\mu$m	Martin et al. (1998)
Hemiosteon radius distribution: standard deviation	$\sigma _{\textrm{On}}$	2.5	$\mu$m	Martin et al. (1998)
Mineral mass density	$\rho _{m}$	3.2	g/cm^3^	Currey (2004)
Water mass density	$\rho _{w}$	1	g/cm^3^	Martin (1984)
Organic matter mass density	$\rho _{o}$	1.1	g/cm^3^	Currey (2004)
Space resolution	$\lambda$	1	$\mathrm {\mu }$m	–
Secondary mineralisation characteristic time	$T_\textrm{sec}$	*f.p.*	years	–
Activation frequency	$\mathrm {Ac.f}$	*f.p.*	BMU/mm^2^ /year	–

#### Estimation of free parameters

**Secondary mineralisation characteristic time**

**Fig. 5:**
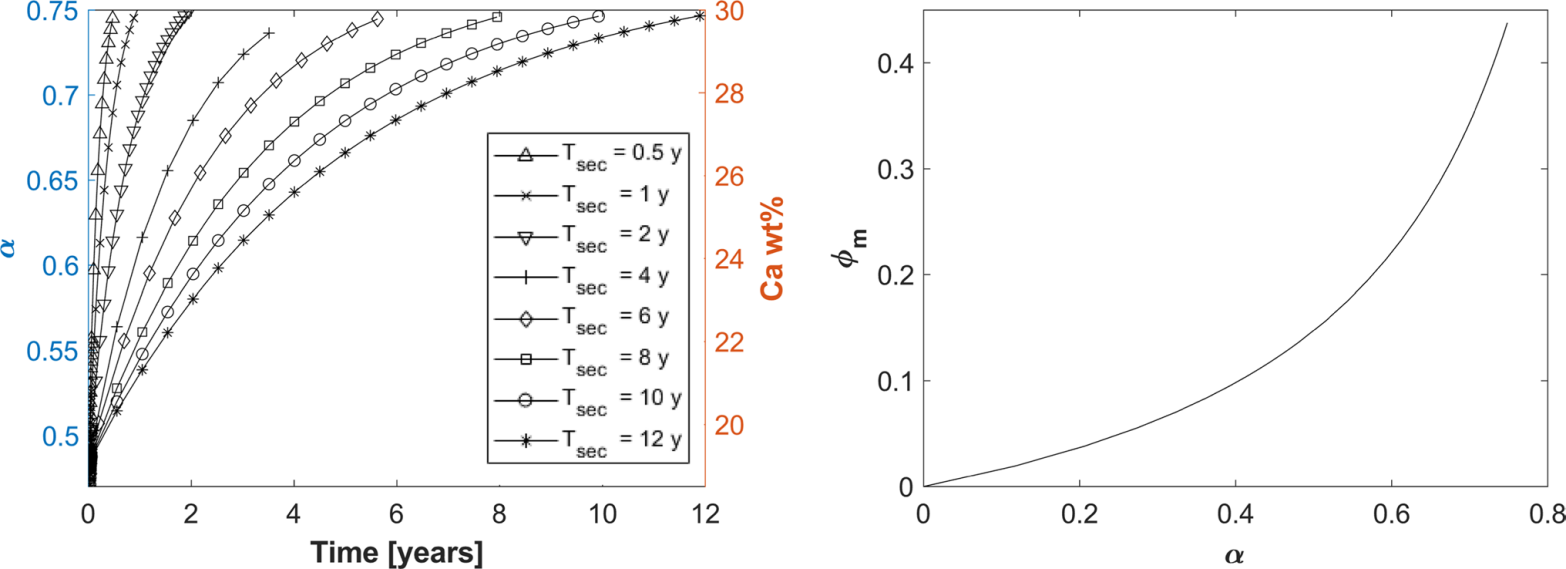
Left panel: Effect of secondary mineralisation characteristic time ($T_\textrm{sec}$) on the ash factor ($\alpha$) and calcium content (Ca wt%). Right panel: Relation between $\alpha$ and mineral fraction ($\phi _{m}$) as described in Eq. (15)

The majority of works agree that the primary mineralisation takes about 5–10 days (Boivin et al. 2009), but discrepancies are found regarding secondary mineralisation time $T_\textrm{sec}$. While some experimental studies state that secondary mineralisation takes place up to several months (Berli et al. 2017), several numerical studies use values of up to 10 years for secondary mineralisation (Martínez-Reina et al. 2008; Bala et al. 2007). Aiming to analyse the mineralisation kinetics, we studied different values for $T_\textrm{sec}$, as seen in left panel of Fig. 5. More details about the secondary mineralisation time $T_\textrm{sec}$ and the parameters of the mineralisation law $\textrm{M}$ in Eq. (14) can be found in Appendix 1.

Even though the mineralisation laws are written in terms of the ash factor $\alpha$, the evolution of mineral fraction over time can be obtained by using Eq. (15). The $\phi _{m}$ in function of $\alpha$ is shown in right panel of Fig. 5.

**Activation frequency**

As $T_\textrm{sec}$, $\mathrm {Ac.f}$ values vary greatly in the literature. On the one hand, Nyman et al considered $\mathrm {Ac.f}=4$ BMU/mm^2^/year for bone remodelling in premenopausal trabecular bone. On the other hand, the experimental study performed by Parfitt (1983) suggests that $\mathrm {Ac.f}$ in trabecular bone is equal to 18 BMU/mm^2^/year. Moreover, values of $\mathrm {Ac.f}$ also vary for pathological cases. Indeed, the unbalance of bone resorption and formation seen in postmenopausal osteoporosis is said to be explained by a rise in $\mathrm {Ac.f}$ (Sambrook and Cooper 2006).

## Results

Given the random nature of the proposed remodelling algorithm, we run fifteen simulations using the same initial geometry, as shown on the left panel of Fig. 9. For each simulation, the random generator seed was initialised based on the current time, resulting in a different sequence of random numbers. Unless otherwise stated, the results are presented in terms of the mean of all the simulations and the parameters presented in Table 2 have been used in the simulations.

### BMDD mean decreases with increase in $\mathrm {Ac.f}$ and $T_\textrm{sec}$

**Fig. 6:**
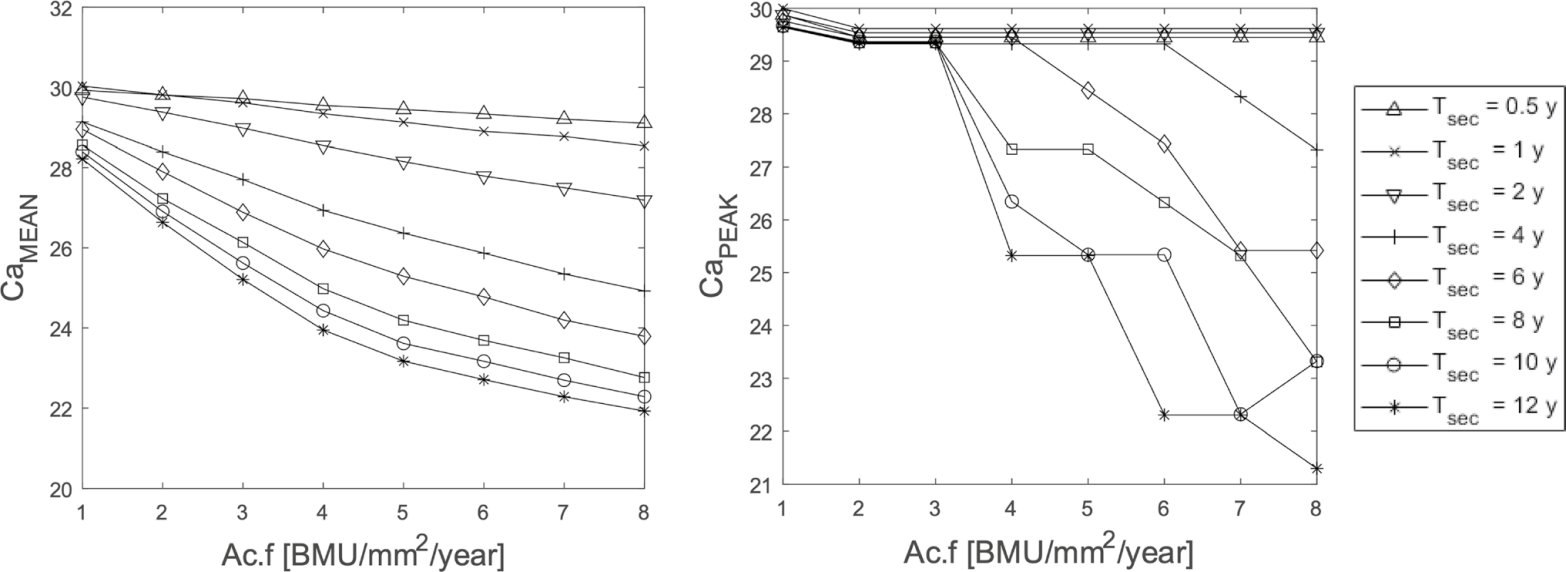
Mean and peak of calcium concentration ($\mathrm {Ca_{MEAN}}$ and $\mathrm {Ca_{PEAK}}$ ) versus $\mathrm {Ac.f}$ for different mineralisation kinetics ($T_\textrm{sec}$), evaluated at simulation time T=5 years

First, we study the effect of $\mathrm {Ac.f}$ and mineralisation kinetics in the healthy remodelling process. Figure 6 presents the average calcium content $\mathrm {Ca_{MEAN}}$ and peak calcium content $\mathrm {Ca_{PEAK}}$, at $T=5$ years for varying activation frequency $\mathrm {Ac.f}$ (ranging from 1 to 8 BMU/mm^2^/year) and secondary mineralisation periods $T_\textrm{sec}$ (ranging from 0.5 to 12 years). It can be noticed that $\mathrm {Ca_{MEAN}}$ decreases as either of $\mathrm {Ac.f}$ or $T_\textrm{sec}$ increases. The standard deviation of $\mathrm {Ca_{MEAN}}$ varies from 0.0012 to 0.0477 wt%, and is therefore not shown. Moreover, the $\mathrm {Ca_{PEAK}}$ only decreases for high values of $\mathrm {Ac.f}$ and slow secondary mineralisation.

**Fig. 7:**
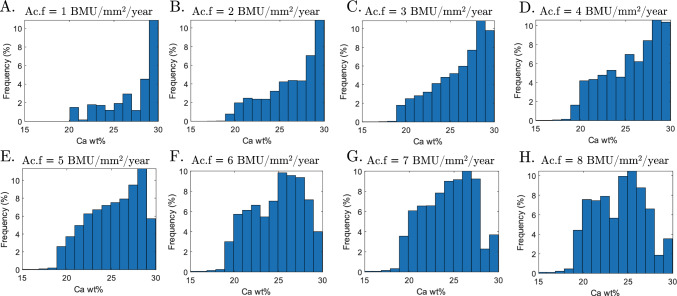
BMDD for mineralisation kinetics $T_\textrm{sec}=8$ years and $\mathrm {Ac.f}$ equal to **A** 1 BMU/mm^2^/year, **B** 2 BMU/mm^2^/year, **C** 3 BMU/mm^2^/year, **D** 4 BMU/mm^2^/year, **E** 5 BMU/mm^2^/year, **F** 6 BMU/mm^2^/year, **G** 7 BMU/mm^2^/year, and **H** 8 BMU/mm^2^/year

**Fig. 8:**
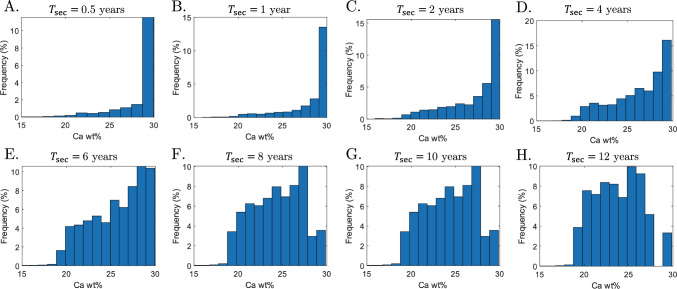
BMDD for an activation frequency $\mathrm {Ac.f}= 4$ BMU/mm^2^/year and mineralisation kinetics $T_\textrm{sec}$ equal to **A** 0.5 years, **B** 1 year, **C** 2 years, **D** 4 years, **E** 6 years, **F** 8 years, **G** 10 years, and **H** 12 years

The effects of $\mathrm {Ac.f}$ and $T_\textrm{sec}$ on the distribution of Ca wt% can be appreciated looking at the variations of the BMDDs with respect to either $\mathrm {Ac.f}$ (Fig. 7) or $T_\textrm{sec}$ (Fig. 8). On the one hand, for a given mineralisation kinetics ($T_\textrm{sec}=8$ years), the BMDD follows a negatively skewed distribution for lower values of $\mathrm {Ac.f}$, and the peak of Ca wt% is about 30% (Fig. 7A–D). On the other hand, skewness of the BMDD decreases with higher values of $\mathrm {Ac.f}$, and the peak of Ca wt% decreases towards 25% (Fig. 7E–H).

A similar pattern is seen for a constant $\mathrm {Ac.f}$ and varying mineralisation kinetics (Fig. 8). Assuming $\mathrm {Ac.f}$ of 4 BMU/mm^2^/year, the BMDD follows a negatively skewed distribution for fast mineralisation kinetics, and the peak of Ca wt% is about 30% (Fig. 8A–D). Skewness of the BMDD decreases with longer times of second mineralisation, with the peak of Ca wt% decreasing towards 26% (see Fig. 8E–F).

**Fig. 9:**
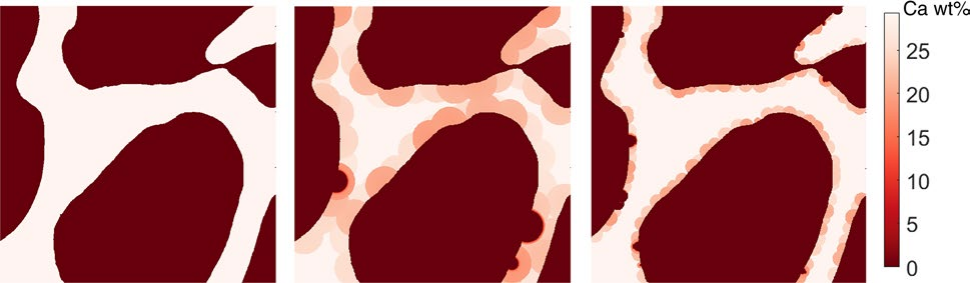
Comparison of BMU formation and resorption spaces from (left) initial geometry to (middle) $R_{\textrm{On}}= 100\mathrm {\mu }$m as in (Martin et al. 1998) and $\mathrm {Ac.f}= 6$ BMU/mm^2^/year and (right) $R_{\textrm{On}}= 40\mathrm {\mu }$m and $\mathrm {Ac.f}=18$ BMU/mm^2^/year as proposed by (Parfitt 1983). Both cases consider $T_\textrm{sec}=8$ years

**Fig. 10:**
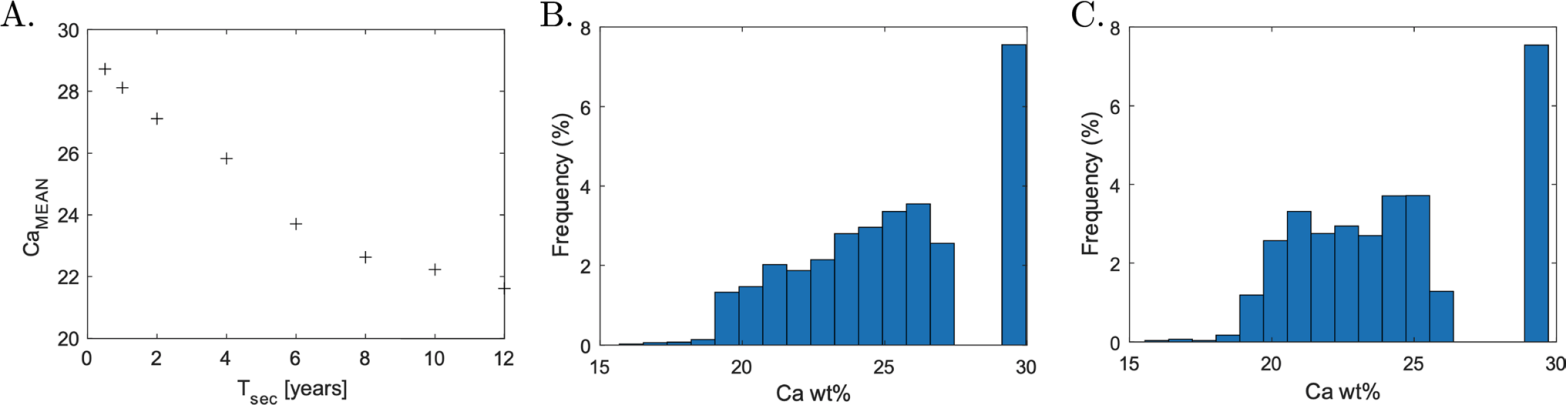
Bone remodelling simulation using the parameters proposed by Parfitt (Parfitt 1983): $R_{\textrm{On}}= 40\mathrm {\mu }$m and $\mathrm {Ac.f}=18$ BMU/mm^2^/year. **A** $\mathrm {Ca_{MEAN}}$ evolution for different mineralisation kinetics. BMDDs for **B** $T_\textrm{sec}=6$ years and **C** $T_\textrm{sec}=8$ years

As mentioned earlier, values of $\mathrm {Ac.f}$ and $T_\textrm{sec}$ vary greatly in the literature. For example, the experimental study performed by Parfitt (1983) suggests that $\mathrm {Ac.f}$ in trabecular bone is equal to 18 BMU/mm^2^/year. We performed five simulations using the suggested parameters ($\mathrm {Ac.f}=18$ BMU/mm^2^/year and $R_{\textrm{On}}= 40\mathrm {\mu }$m). The initial geometry of the simulation is shown in the left panel of Fig. 9; the solutions obtained at $T=8$ years using typical values used in our analysis and those suggested by Parfitt are shown in the middle and right panels of Fig. 9, respectively. The latter simulation shows large areas of interstitial bone that are never remodelled. A deeper insight on the BMDD obtained using the new parameters is shown in Fig. 10. Figure 10A shows the variation of the mean calcium content $\mathrm {Ca_{MEAN}}$ with respect to the secondary mineralisation time $T_\textrm{sec}$. Values of $\mathrm {Ca_{MEAN}}$ between 22% and 24% have been obtained for slower mineralisation kinetics, which are in accordance with $\mathrm {Ca_{MEAN}}$ values seen in the literature (Lerebours et al. 2020). BMDD plots obtained for $T_\textrm{sec}= 6$ years and 8 years are shown in Fig. 10B and C, respectively. BMDDs are negatively skewed and the skewness decreases for slower mineralisation kinetics. Moreover, the frequency peaks at the right of the BMDDs (Ca wt% = 30%) represent bone that is never remodelled in the presented simulations.

### Bone density as function of $\mathrm {Ac.f}$ and $T_\textrm{sec}$

**Fig. 11:**
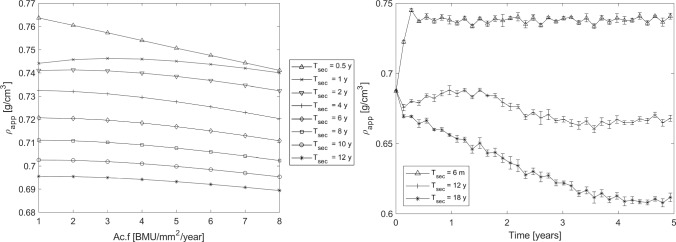
*Left panel*: Apparent density $\rho _{app}$ versus $\mathrm {Ac.f}$ for different mineralisation kinetics ($T_\textrm{sec}$), evaluated at simulation time $T=5$ years. *Right panel*: Example of $\rho _{app}$ evolution for $\mathrm {Ac.f}=4$ BMU/mm^2^/year with $T_\textrm{sec}$ equal to 6 months, 4 years, and 12 years. Data are expressed as mean ± standard deviation

We also analysed the variations of the apparent density $\rho _{app}$ with respect to the activation frequency ($\mathrm {Ac.f}$ ranging from 1 to 8 BMU/mm^2^/year) and mineralisation kinetics ($T_\textrm{sec}$ ranging from 0.5 to 12 years)–see Fig. 11. As for $\mathrm {Ca_{MEAN}}$, results in Fig. 11 (left panel) show that $\rho _{app}$ decreases as either $\mathrm {Ac.f}$ or $T_\textrm{sec}$ increases. On the other way round, low bone turnover combined with faster secondary mineralisation results in higher densities.

As shown in Fig. 11 (right panel), for a given $\mathrm {Ac.f}$, there is a mineralisation kinetics that maintain a constant $\rho _{app}$ through the remodelling process. For example, a combination of $\mathrm {Ac.f}=4$ BMU/mm^2^/year and $T_\textrm{sec}= 4$ years results in small variations of $\rho _{app}$ that remains about 0.68 g/cm^3^.

### Bone remodelling and osteoporosis

**Fig. 12:**
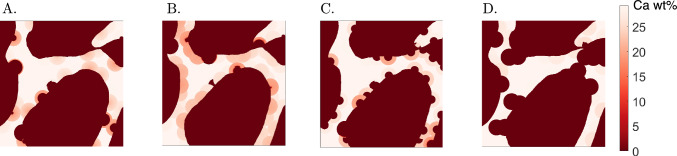
Bone microstructure observed for different simulations of osteoporosis: **A** *High* $\textrm{FP}$ scenario, corresponding to $\textrm{FP}=475$ days (Villanueva et al. 1966). **B** *High* $\mathrm {Ac.f}$ scenario, corresponding to $\mathrm {Ac.f}= 8$ BMU/mm^2^/year. **C** *Underfilling* scenario, corresponding to partial refilling of the resorption cavities, with $u_{\textrm{f}}= 50\,\%$. **D** *No filling* scenario, corresponding to no refilling of the resorption cavities, *i.e.* $u_{\textrm{f}}= 100\,\%$. Simulation time: $T=3$ years

Finally, we simulate different types of primary osteoporosis, such as post-menopausal (type I) and senile (type II) osteoporosis. From a biological point a view, post-menopausal osteoporosis is associated with an unbalanced activity of osteoclasts and osteoblasts, namely when bone resorption prevails on bone formation. This imbalance can be magnified by a rise in $\mathrm {Ac.f}$ (Sambrook and Cooper 2006). Therefore, we simulate type I osteoporosis following the hypothesis that it is related to longer formation periods $\textrm{FP}$ (Villanueva et al. 1966) or to high $\mathrm {Ac.f}$ (Sambrook and Cooper 2006) (Fig. 12A and B, respectively). On the other hand, senile osteoporosis is mainly characterised by a shift from osteoblastogenesis to predominant adipogenesis in the bone marrow that results in a reduction in bone formation (Duque and Troen 2008). Accordingly, we describe senile osteoporosis following the hypothesis that it occurs through an underfilling of semiosteons or that no formation takes place, which is usually named as uncoupling (Delaisse et al. 2020) (Fig. 12C and D, respectively).

**Fig. 13:**
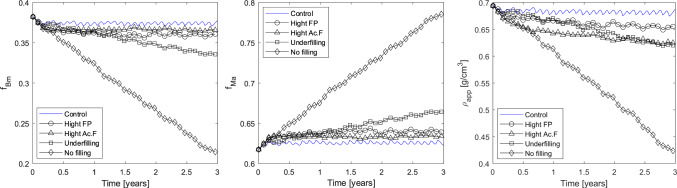
Evolution of bone matrix $f_{\textrm{Bm}}$ (left) and marrow $f_{\textrm{Ma}}$ (middle) fractions, and apparent density $\rho _{app}$ (right) for the different osteoporosis simulations and a healthy simulation (control)

Since osteoporosis is characterised by a low bone mass and micro-architectural deterioration associated with a negative net balance between bone formation and resorption during bone remodelling (Langdahl et al. 2016), we analysed the evolution of the bone matrix ($f_{\textrm{Bm}}$) and marrow ($f_{\textrm{Ma}}$) fractions, and of the apparent density ($\rho _{app}$). We first performed a simulation in physiological conditions ($\mathrm {Ac.f}=4$ BMU/mm^2^/year and $T_\textrm{sec}=4$ years). The configuration obtained after 5 years of physiological remodelling was used as initial configuration for the simulations describing pathological remodelling. We simulated different scenarios leading to osteoporotic bone, by modifying the parameters used for physiological remodelling: the *High* $\textrm{FP}$ scenario corresponds to $\textrm{FP}=475$ days (Villanueva et al. 1966); the *High* $\mathrm {Ac.f}$ scenario corresponds to $\mathrm {Ac.f}= 8$ BMU/mm^2^/year; the *Underfilling* scenario corresponds to $u_{\textrm{f}}= 50\,\%$; and the *No filling* scenario corresponds to $u_{\textrm{f}}=1$. *High* $\textrm{FP}$ and *High* $\mathrm {Ac.f}$ scenarios refer to postmenopausal osteoporosis. Scenarios *Underfilling* and *No filling* scenarios refer to senile osteoporosis. All these simulations describe the evolution of bone microstructure over 3 years. All results are compared to a control case, corresponding to a 3-year physiological remodelling. The time course of $f_{\textrm{Bm}}$, $f_{\textrm{Ma}}$, and $\rho _{app}$ are shown in Fig. 13.

When the formation phase is prevented and BMUs are not filled (*No filling* scenario), a drastic loss of bone mass and an important decrease in $\rho _{app}$ can be observed. This is a severe case of osteoporosis where the microstrucure is severely affected (Fig. 12D), and the bone recovery is extremely unlikely, since it may include perforation of trabeculae. For the other cases, variations in bone matrix fraction and apparent density are less important, but still noticeable when compared to the control. The *Underfilling* scenario shows a marked decrease of both bone matrix fraction $f_{\textrm{Bm}}$ and apparent density $\rho _{app}$. Both *High* $\mathrm {Ac.f}$ and *High* $\textrm{FP}$ scenarios show a limited decrease of $f_{\textrm{Bm}}$. However, the latter show a pronounced decrease of $\rho _{app}$, comparable to that of the *Underfilling* scenario.

**Fig. 14:**
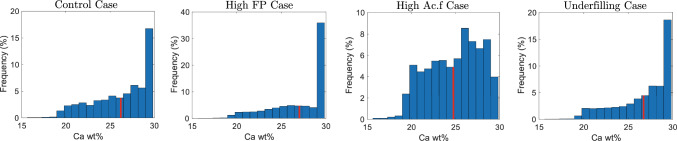
BMDDs in control (left: *Control* scenario), postmenopausal osteopororis (middle left: *High* $\textrm{FP}$ scenario; middle right: *High* $\mathrm {Ac.f}$ scenario), and senile osteoporosis cases (right: *Underfilling* scenario). $\mathrm {Ca_{MEAN}}$ is represented in red

When looking at the BMDD, it can be observed that the $\mathrm {Ca_{MEAN}}$ decreases for the postmenopausal case with high $\mathrm {Ac.f}$ (see Fig. 14 left). This decrease is expected, since an increase in $\mathrm {Ac.f}$ results in a less mineralised tissue. Our numerical results agree with experimental observations that show a shift of the BMDD towards lower values of Ca content in postmenopausal cases (Zoehrer et al. 2006) (see also Fig. 1). Furthermore, for senile osteoporosis, the Ca concentration has a tendency to increase, since little to no bone is being formed while bone continues to mineralise (Fig. 14 right).

## Discussion

The current model considers the process of mineralisation of the bone matrix, differently than existing discrete remodelling models, such as the ones proposed by Martin (1984, 1991); Nyman et al. (2004). As discussed by many authors, computational models that neglect bone matrix mineralisation are not able to predict increases in apparent bone density (or intrinsic bone density) for cases when bone remodelling is significantly reduced, such as for example in the use of anti-resorptive drugs (Scheiner et al. 2014; Martínez-Reina and Pivonka 2019).

Values used for the activation frequency $\mathrm {Ac.f}$ in our numerical simulations are lower than values suggested by some experimental studies (Parfitt 1983). These lower values could be because the width of hemiosteons may vary with the geometry of trabeculae. If hemiosteons are smaller, a higher $\mathrm {Ac.f}$ is needed to achieve the same bone turnover. The value of 18 BMU/mm^2^/year was proposed by Parfitt (1983) for hemiosteons having a radius of $40\mathrm {\mu }$m. In our simulations, we used a hemiosteon radius $R_{\textrm{On}}=100\mathrm {\mu }$m. Therefore, $\mathrm {Ac.f}$ should be considerably smaller, as used in the current simulations, which agrees with the values of $\mathrm {Ac.f}$ used by Nyman et al. (2004).

We found that BMDD depends both on $\mathrm {Ac.f}$ and $T_\textrm{sec}$ as shown in Figs. 7, 8. This finding supports the conceptual model of Boivin and Meunier (2002, 2003) which suggests these two factors to affect the BMDD. Similar trends in BMDD were found by Ruffoni et al. (2007) et al using the continuous BMDD balance approach. However, unlike in Ruffoni et al. (2007), the BMDD histograms of our simulations were not sensitive to the type of mineralisation curve (*i.e. *hyperbolic versus exponential) as long as $T_\textrm{sec}$ and the maximum level of mineralisation were kept similar.

For situations where secondary mineralisation is fast (*i.e.* $T_\textrm{sec}$ equal to one year or below), most of bone is highly mineralised, and this leads to a negatively skewed BMDD histogram (Fig. 8A–B). Therefore, although Martin (1991) states that variation in $\mathrm {Ac.f}$ alone can be responsible for bone remodelling an define bone gain and loss, the mineralisation kinetics play an essential role in determining the BMDD. In accordance with Lerebours et al. (2020), we found that $\mathrm {Ac.f}$ and $T_\textrm{sec}$ have a coupled effect on the BMDD.

For lower activation frequency or fast secondary mineralisation, the BMDDs are strongly negatively skewed. Skewness of BMDDs decreases for higher activation frequency or slow secondary mineralisation, as observed through quantitative backscattered electron imaging (qBEI) (Roschger et al. 1998). qBEI experimental results on trabecular bone suggest values of $\mathrm {Ca_{MEAN}}$ ranging between 22 and 24 wt% (Lerebours et al. 2020). Our numerical results show a $\mathrm {Ca_{MEAN}}$ slightly higher (between 24 and 26 %). This could be explained by some approximations of the model. Firstly, only a two-dimensional slice of trabecular bone was considered, which leads to some inner trabecular regions having never been remodelled, because BMUs are activated on bone surfaces only. Secondly, BMU activation in the bulk of the trabeculae are not considered by our model. However, bulk remodelling within trabeculae–creating cylindrical osteons similar to those observed in cortical bone–does occur (Sato et al. 1986), and could serve as a means of targeting areas of higher Ca content within thicker trabeculae.

Unlike $\mathrm {Ca_{MEAN}}$, the apparent bone density only changes slightly for different $\mathrm {Ac.f}$ and $T_\textrm{sec}$. In the studies cases, $\rho _{app}$ varies from 0.6 to 0.75 g/cm^3^, which agrees with density values of trabecular bone seen in the literature (Zioupos et al. 2008; Öhman-Mägi et al. 2021)

The osteoporotic remodelling patterns highlight that even though the apparent density for type I and type II osteoporosis is comparable, the former is due to a high percentage of newly formed bone, while the latter is due to general bone loss. Therefore, our results suggest that analysing only the apparent bone density is not be sufficient to understand the nature of bone pathology. Moreover, besides presenting a lower $\rho _{app}$, the case with higher $\mathrm {Ac.f}$ also shows a shift in $\mathrm {Ca_{MEAN}}$, as seen experimentally (Zoehrer et al. 2006). These results suggest that type I osteoporosis is mainly due to high $\mathrm {Ac.f}$ rather than longer $\textrm{FP}$.

Finally, our study has a number of limitations. Firstly, no mechanical loading has been included in our simulations. However, mechanical loading has been shown to potentially affect mineralisation kinetics (Tourolle né Betts et al. 2020). Also, mechanical loading may induce microcracks in the bone matrix which could serve as a target for BMU initiation. Here, we used higher mineralised bone tissue as a target for BMU activation. Secondly, BMU resorption, reversal and formation events were assumed to vary linearly, and were implemented in an *ad hoc* fashion with no biological mechanism considered. Lastly, trabecular BMUs were described by Parfitt (1994) as spatio-temporal systems where osteoblasts follow osteoclasts as they progress along the bone surface. This was depicted as equivalent to half a cortical BMU and thus entailing a trench-like pattern called hemiosteons. Similarly, in a recent review, Delaisse et al. (2020) depict a trabecular BMU as progressing and creating a “resorption track”. Our modelling is not inconsistent with trench-like BMUs progressing perpendicularly to the 2D cross section of trabecular bone considered in our study, leading to semi-circular patterns in the cross section. However, it cannot describe BMUs progressing on in other directions, namely along the bone surface in the plane of the cross section. With respect to the cross-sectional shape of trabecular BMUs, a recent histological 2D study of osteopontin-stained cement lines in human vertebral samples, revealed a lens-shaped morphology, deepest in the centre and progressively thinner towards their peripheries (Lamarche et al. 2022). While trabecular remodelling has been extensively studied, there is relatively little direct 3D morphological BMU data published. Serial block face imaging revealed trabecular remodelling events characterised by indentations on trabecular surfaces, which is relatively similar to the idealised forms we modelled (Slyfield et al. 2012; Tkachenko et al. 2009). However, more extended trench-like forms have also been reported (Tkachenko et al. 2009). Kragstrup and Melsen (1983) described trabecular BMUs from human samples as “broad bands” or “plate shaped” with extended trench-like structures, rather than punctate forms. Furthermore they observed that hemiosteons could be curved, branched and even cylindrical, which is not taken into account in our work and should be studied in future works.

The ultimate aim of our modelling approach is to also add mechanics to this model. For instance, introducing information about dynamics of loading cycles and material behaviour may allow to investigate the coupling between BMDD and stress patterns in the bone. In turn, this will allow to analyse and track accumulation of microcracks in the bone matrix, and ultimately estimate risk of bone fracture, which would depend on BMDD.

## Conclusions

In this work, we propose a discrete statistical model of BMU remodelling in trabecular bone. Besides its geometrical algorithmic nature, this discrete model allows the study of the evolution of bone microstructure, apparent bone density, and bone mineral density distribution as a function of BMU activation frequency and time of secondary mineralisation. The major findings of our numerical simulations are:

- BMDD is strongly influenced both by activation frequency of BMUs and mineralisation kinetics.
- Normal BMDD histograms for healthy equilibrium bone remodelling situations are obtained for different combinations of $\mathrm {Ac.f}$, and $T_\textrm{sec}$ values.
- For equilibrium cases $\mathrm {Ca_{MEAN}}$ depends on the value of $\mathrm {Ac.f}$ with higher $\mathrm {Ac.f}$ giving lower $\mathrm {Ca_{MEAN}}$ values.
- Apparent bone density and BMDD should be mutually analysed to understand the nature of bone diseases, especially osteoporosis.

Future model developments will include mechanical aspects of bone remodelling and mechanobiological regulation of BMUs.

## Figures and Tables

**Fig. 15:**
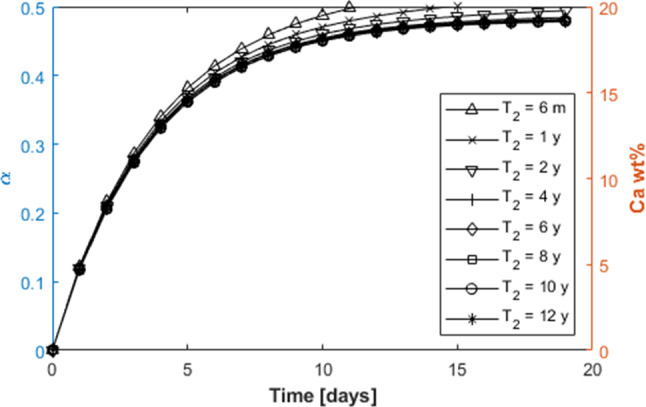
Short time scales highlight the first mineralisation kinetics. The evolution of the ash factor is little affected by the secondary mineralisation time $T_\textrm{sec}$

## Appendix A Mineralisation law parameters

Table 3 presents the parameters for the double-exponential mineralisation law described in Eq. (14) for different secondary mineralisation kinetics. Note that the only varying parameter is the characteristic time $t_1$, which is mainly responsible for the “slope” of the second exponential curve, *i.e. *the secondary mineralisation. By contrast, this parameter does not affect noticeably the first mineralisation–see Fig. 15. The progress of second mineralisation with respect to $T_\textrm{sec}$ is shown in Fig. 5.

**Table 3:** Mineralisation law parameters for different secondary mineralisation times $T_\textrm{sec}$

$T_\textrm{sec}$ [years]	$c_1$ [Ca wt%]	$c_{\textrm{max}}$ [Ca wt%]	$t_1$ [days]	$t_2$ [days]
6 months	11.9	31	75	3.5
1 year	11.9	31	140	3.5
2 years	11.9	31	290	3.5
4 years	11.9	31	630	3.5
6 years	11.9	31	900	3.5
8 years	11.9	31	1250	3.5
10 years	11.9	31	1550	3.5
12 years	11.9	31	1850	3.5
